# Disentangling Language Disorder and Bilingualism in Children with Developmental Language Disorder and Autism Spectrum Disorder: Evidence from Writing

**DOI:** 10.1007/s10803-022-05727-4

**Published:** 2022-09-10

**Authors:** Eleni Peristeri, Ianthi Maria Tsimpli

**Affiliations:** 1 School of English, Faculty of Philosophy, Aristotle University of Thessaloniki, 541 24 Thessaloniki, Greece; 2 Faculty of Modern and Medieval Languages and Linguistics, University of Cambridge, Cambridge, English Faculty Building, Room TR-11, 9 West Road, Cambridge, CB3 9DP UK

**Keywords:** Bilingualism, Writing, Autism spectrum disorder, Developmental language disorder, Spelling, Punctuation

## Abstract

Twenty-eight Albanian-Greek bilingual children with Developmental Language Disorder and 28 children with Autism Spectrum Disorder but no language impairment, along with 28 typically-developing, age-, Performance IQ- and socioeconomic status-matched bilingual children were asked to produce two expository texts which were coded for spelling (phonological, grammatical, orthographic) errors, stress and punctuation use. The children’s expressive vocabulary, current language use and home language history were also measured. The results show that the bilingual children with Developmental Language Disorder were particularly vulnerable to spelling errors, while their bilingual peers with Autism Spectrum Disorder were rather challenged by stress and punctuation. The evidence speaks in favor of distinct patterns of writing impairment across the bilingual children with Developmental Language Disorder and Autism Spectrum Disorder.

There is an emerging body of research on the acquisition of conventional literacy skills, including writing, in children with neurodevelopmental disorders, including Developmental Language Disorder (DLD) and Autism Spectrum Disorder (ASD). Written text generation in both populations has been investigated in terms of code-related, or else transcription aspects of forming written representations of words, such as letter-sound correspondences, orthographic knowledge and stress assignment (e.g., Broc et al., 2013; Critten et al., 2014 for children with DLD; Cardoso-Martins et al., 2015; Wiggins et al., 2010 for children with ASD), as well as meaning-related processes, including content selection, lexical choices and appropriate transitions between sentences, among others (e.g. Dockrell & Connelly, 2015; Favart et al., 2016 for children with DLD; Brown & Klein, 2011; Brown et al., 2014 for children with ASD). The current study focuses on children’s code-related skills during expository text generation, so henceforth the term writing will be used to refer to the particular component of written production.

Though the aforementioned studies acknowledge that children with DLD and ASD are at risk for poor code-related writing skills, no evidence exists on the way dual language exposure affects the specific populations’ writing performance. As researchers try to cope with challenges that bilingualism poses for the diagnosis and treatment of children with neurodevelopmental disorders (e.g. Beauchamp & MacLeod, 2017), further information is necessary to characterize the writing abilities of bilingual children with DLD and ASD. For these reasons, the current study aims to examine the expository texts constructed by bilingual children with DLD and ASD, as well as age-, Performance IQ (PIQ)-, and socioeconomic status (SES)-matched typically-developing (TD) bilingual children, in terms of their spelling and word stress assignment skills, as well as the use of punctuation marks whose role in writing extends beyond supporting the physical segmentation of text. The present study also examines how children’s language ability and bilingual status contribute to their writing performance. The data of the current study have been collected from autistic children living in Greece, where the ASD prevalence in 2019 was 1.2% (Thomaidis et al., 2020).

The rest of the Introduction will review the literature on the writing performance of TD, DLD and ASD monolingual children, with an emphasis on their spelling skills, the use of stress and punctuation, in order to highlight where difficulties in the specific writing components are known to occur in the two disorders. We next review the literature on the effects of bilingualism on the language abilities of children with DLD and ASD, since understanding the language skills of these groups is critical to allow us to provide an explanation of their writing deficits in terms of their language profile. Finally, we provide an overview of the orthographic and stress assignment properties of the Greek language, in which all the tasks of the current study were administered, as well as information on the developmental trajectory of spelling, stress and punctuation use in TD monolingual Greek-speaking children.

## Writing Ability in TD Bilingual Children

Research in second language (L2) writing demonstrates that bilingual children draw on their native language to fulfil writing requirements, with the strength of cross-language transfer being modulated by the length of the children’s L2 exposure and proficiency (see Williams & Lowrance-Faulhaber, 2018 for a review). Cross-linguistic influence has been found to be particularly strong for spelling as well as orthography-specific skills during handwritten production in the L2, especially in children speaking alphabetic languages in which phonological as well as orthographic overlap boosts transfer of spelling strategies across the two languages (Sun-Alperin & Wang, 2011; Yeong et al., 2014). The majority of the studies found that the bilingual groups exhibit weaker spelling skills in the L2 than their TD monolingual peers (e.g. Caravolas et al., 2020; Guimaraes & Parkins, 2019; Li et al., 2012a, 2012b), even when monolingual and bilingual children have comparable amounts of writing experience in their main language of instruction (Caravolas et al., 2020). Spelling errors in both Caravolas et al.’s (2020) and Li et al’s. (2012a, 2012b) studies did not differ across the children’s L1 and L2, while word spelling ability in L1 emerged as a significant predictor of spelling skills in the L2, thus, suggesting transfer of orthographic properties across the two languages.

While most work investigating the writing development of TD bilingual children has focused on their code-related (i.e. letter knowledge, spelling) skills, very little attention has been paid to the direct empirical investigation of prosodic patterns underlying the use of punctuation in bilingual children’s written texts. There are studies with monolingual adults that have mainly focused on the potent disambiguating role that punctuation may play in the processing of locally ambiguous sentences using self-paced reading paradigms (Drury et al., 2016; Niikuni & Muramoto, 2014; Steinhauer & Friederici, 2001). These studies converge to show that punctuation marks (especially commas) play a critical role in cueing types of parsing decision at particular points in sentences with local processing difficulty; in a sense, they guide readers through surface text organization by facilitating written text chunking into coherent blocks and pulling the interpretation of separate punctuation-marked blocks towards a globally coherent parse. Punctuation marks in written production may thus be regarded as visual markers of coherent syntactic and conceptual dependencies that would otherwise be unrelated. In this sense, they may provide the same facilitatory benefits in chunking written discourse as explicit prosody (i.e. the intonation patterns of pitch accents and phrase boundaries and rhythmic timing) provides during listening comprehension, at least in neurotypical individuals (Steinhauer, 2003). Indeed, in Heggie and Wade-Woolley’s (2018) study, adults with high prosodic awareness skills in oral language were more efficient to correctly apply punctuation in written speech, over and above the influence of their baseline punctuation knowledge, as compared to their peers with lower prosodic awareness skills. Similarly, Calet et al. (2017) found that TD children that have been trained on manipulating expressive prosody to encode information structure in a reading-aloud task tended to perform more accurately than untrained children in a written prosody task, in which they had to insert punctuation marks in unpunctuated sentences in order to assign to them a particular meaning. The overall findings support the hypothesis that the ability to use prosodic ‘boundaries’ to capture the information structure of sentences in oral speech may have important consequences for the appropriate use of punctuation marks in writing, which may, in turn, reflect an implicit or internal prosodic representation, also known as implicit prosody (Fodor, 2002). The way TD bilingual children, as well as bilingual children with ASD and DLD use implicit prosodic cues while conveying meaning in text production is considerably underexplored.

## Writing Ability in Monolingual Children with DLD and ASD

Though the effects of DLD and ASD on the oral language abilities of school-aged monolingual and bilingual children have been extensively reported across various methodological designs and languages (e.g. Broc et al., 2021; Durrleman & Delage, 2016; Goldman, 2008; Huang & Finestack, 2020; Peristeri et al., 2017; Schaeffer et al., 2014, among many others), writing skill in the two disorders, especially autism, has received relatively little attention.

The effect of the disorder on the writing skills of monolingual children with DLD is declared to be robust. Especially, spelling has been shown to be a particularly vulnerable domain for children with DLD, who seem to fall behind their age-matched peers on applying orthographic and morphological spelling conventions (Brizzolara et al., 2011; Broc et al., 2013; Cordewener et al., 2012; Critten et al., 2014; Larkin et al., 2013). According to Graham et al.’s (2020) recent meta-analysis, when language was controlled across studies, DLD children’ s spelling difficulties were significantly greater than their language-matched peers. Crucially, a number of studies (e.g. Dockrell & Connelly, 2015; Dockrell et al., 2007, 2009; Mackie et al., 2013; Puranik & Lonigan, 2012) have demonstrated a strong influence of DLD children’s oral language ability (i.e. expressive vocabulary, phonological awareness, recalling sentences, oral speech perception) on their spelling skills. These findings indicate that oral language difficulties in monolingual children with DLD can drive their spelling performance in written text production. These results also align well with established findings in relevant research in TD monolingual children that spelling is backed up by the child’s phonological awareness skills which become more strengthened as breadth of expressive vocabulary increases in order to avoid confusion of lexical items having similar phonological representations (Cassano & Schickedanz, 2015; Metsala, 1999). At the same time, these findings add to our growing understanding of how code-related aspects of writing, and especially spelling, in DLD may be affected by the children’s oral language difficulties.

Studies on the writing skills of monolingual individuals with ASD have been considerably fewer relative to children with DLD, and reveal asymmetries across code- and meaning-related aspects of writing. Specifically, at the level of written word representation, monolingual autistic children and adults have been found to exhibit poor handwriting legibility and letter formation (Kushki et al., 2011), while their performance at the macro-organizational level of writing is characterized by poor global and local coherence relations between the ideas (Brown & Klein, 2011; Dockrell et al., 2014). On the other hand, there is evidence showing that syntactic complexity and lexical diversity in written production are well-preserved in autism, and that autistic children’s rates of spelling mistakes are comparable to those in TD children (Hilvert, 2018). While there is no robust support to link ASD children’s spelling performance to their oral language skills, there has been some evidence for a correlation between word spelling accuracy and phonological awareness skills, but not oral vocabulary knowledge (Bailey & Arciuli, 2018). Phonological awareness has been found to be an important emergent literacy predictor for kindergarten children with ASD (Dynia et al., 2017), and it has been associated with their weak sound-to-letter mappings. On the other hand, vocabulary knowledge appears to be a preserved component of autistic children’s oral language competency, and even seems to be boosted by the autistic condition (e.g. Davidson, 2021; Kissine et al., 2019; Durrleman et al., 2022).

Furthermore, monolingual children with ASD have been reported to differ from TD peers on explicit prosodic properties of their speech. In fact, deficits in speech prosody in individuals with ASD are one of the earliest symptoms of the disorder (Tager-Flusberg et al., 2005) and are reported to persist even if other areas of language improve (McCann & Peppé, 2003). Prosodic impairments in ASD have been mainly observed in language production, and more specifically, in the use of prosody to convey phrase-level stress (McCann et al., 2007), in slow syllabic speech (Baron-Cohen & Staunton, 1994) and in increased pitch range in conversation (Nadig & Shaw, 2012). The functional role of prosody in perception has been found to be relatively more preserved than production, since monolingual children with ASD have been shown to discriminate word pairs with distinct lexical stress patterns (Grossman et al., 2010), to recall better stressed than unstressed words (Fine et al., 1991), and to distinguish between sentence types, such as statements and questions (Paul et al., 2005; Peppé et al., 2007, 2011). Interestingly, Geelhand et al. (2021) have recently investigated syntax-prosody mappings in the speech production of monolingual autistic and neurotypical adults, and found that the two groups differed in the strategies they employed to combine syntactic and prosodic information; crucially, the autistic adults were reported to condense significantly more syntactic units into a single prosodic unit (delineated by silent pause) relative to neurotypical adults who were found to use considerably more discourse markers (i.e. lexemes that serve a structuring function, e.g., *well*, *you know*, *I mean*) than the autistic group. Geelhand et al. (2021) took this evidence to suggest that explicit prosody in the delivery of oral speech can distinguish autistic adults’ discourse coherence management strategies from their neurotypical peers. Differences in speech prosody perception have also been identified as a characteristic of children with DLD. For example, Richards and Goswami (2015) have found that monolingual children with DLD fall behind their TD peers on stress perception at both the lexical and phrasal level, while Cumming et al., (2015) show that children with DLD face general perceptual difficulties with the global prosodic structure of spoken language. Though prosodic deficits in spoken language have been the focus of research in populations with DLD and ASD, the way these deficits map into the use of punctuation marks in written speech production in the same populations is as of yet not well understood.

## Bilingualism Effects in Children with Language Disorder

The existing literature on the language development of bilingual children with DLD and ASD suggests that bilingualism does not seem to have a negative impact on the language abilities of these children, who can successfully acquire two languages. Studies indicate that, when provided with adequate language exposure, bilingual children with ASD develop language similar to their monolingual non-verbal IQ matched peers (Beauchamp et al., 2020; Gonzalez- Barrerro & Nadig, 2018, 2019; Lund et al., 2017). In addition, Dai et al., (2018) found that, though bilingual children with ASD showed greater language deficits than their bilingual peers with DLD, there was no adverse effect of bilingual exposure on any of the groups’ language performance. Bilinguals and monolinguals have been found to perform comparably on various language tasks (e.g., Drysdale et al., 2015; Meir & Novogrodsky, 2019, for children with ASD; Paradis et al., 2003, 2022; Schwob & Skoruppa, 2022; Tsimpli et al., 2016 for children with DLD), while it is often the case that lower performance in bilinguals for vocabulary can be attributed to the fact that this measure has been only administered in a single language and thus does not represent a bilingual’s total lexicon (Bedore & Peña, 2008). Previous work on ASD, specifically, has revealed that bilinguals who master fewer words than their monolingual peers when the lexicon is measured in a single language in fact have a larger total number of words when this is measured across both languages (Petersen et al., 2012). Beauchamp and MacLeod (2017) have stressed the urgent need to sensitize professionals to existing literature, and to formulate guidelines to assist the decision-making process for selecting language(s) for exposure in children with ASD growing up in bi/multilingual environments. To the best of our knowledge, no study has investigated the written text production of bilingual children with ASD and DLD, or the contribution of vocabulary to bilingual DLD and ASD children’s writing performance.

## Writing Development in Greek

Since writing performance in the current study was assessed in the bilingual children’s L2/Greek, the current section presents an overview of the orthographic and stress assignment properties of the Greek language, as well as information on the developmental trajectory of spelling, stress and punctuation use in TD monolingual Greek-speaking children.

Greek is considered to be an orthographically transparent language. Specifically, grapheme-to-phoneme consistency is around 80% in the sound-to-spelling direction (Protopapas & Vlahou, 2009), with graphemes representing either single letters or letter combinations that behave like single letters, for example the ει/i/ in Greek. Phoneme-to-grapheme mappings in Greek are more transparent than English, yet, less transparent than Hungarian, Dutch and German. The moderate transparency of the Greek orthographic system mainly stems from irregularities in vowel-to-grapheme mappings. For instance, the vowel phoneme /ε/ is represented by the graphemes αι and ε, while the vowel phoneme /ι/ is graphically represented by η, ι, υ, οι, ει, and υι. Vowel spelling irregularities are the main factor influencing Greek-speaking children’s spelling performance, since the overwhelming majority of spelling errors consist of selections of mutually exclusive graphemes that give raise to phonologically acceptable representations (e.g. target: γράφει/γrafi/“(s/he) writes—spelling error γράφι/γrafi/“(s/he) writes; the spelling error is underlined” (Diamanti et al., 2014; Prootopapas et al., 2013).

Since orthographic representations in Greek arise as a by-product of relatively consistent sound-spelling mappings, Greek-speaking children develop phonological strategies, i.e. they tend to rely on sound-letter correspondences, at the phoneme and syllable level at relatively early stages of literacy development, specifically at the age of 5 years (Porpodas, 2001; Protopapas et al., 2013). For instance, the word γυναίκα/γineka/“(woman) is frequently misspelled as γινέκα, implying that children fail to consistently integrate orthographic information at this developmental stage. The repertoire of strategies to support spelling abilities is then gradually expanded to include word-specific orthographic information as well as morphological knowledge mainly related to the inflectional endings of various grammatical categories, e.g. active voice verbs are composed of the stem and the inflectional ending —ω/o/, which signifies first person singular, while feminine nouns are composed of the stem and the inflectional ending –ες/es/, which signifies plural nominative case. Inflectional types play a critical role for the correct spelling of the inflectional endings (also known as suffixes) in verbs, nouns, adjectives and pronouns (Grigorakis & Manolitsis, 2016; Protopapas, 2017; Tijms et al., 2020), so knowledge of morphological rules allows the child to capture the spelling of word forms in a fine-grained manner (Diakogiorgi et al., 2006; Nunes et al., 2006). In fact, Diamanti et al.’s (2017) study has shown that Greek children as young as 5 years can be sensitive to the grammatical role of word parts, and that they are able to use this morphological information to cope with the spelling demands of Greek suffixes introduced at later grades. Taken together, research on the time-course of the spelling development of Greek-speaking children suggests a feed-forward relationship between phonology and grammar in spelling: the phoneme-to-grapheme correspondences are retrieved, and then the phonological strategies combine with suffixes subject to a set of well-formedness principles, overridden only by lexically-specific information. Convergence to the final stage of appropriate spelling takes place at the age of 11 years (Georgiou et al., 2008). This is in line with the objectives of the official programs of studies in primary education, as well as school books, which have been recently updated by the Greek Institute of Educational Policy in Greece. By the age of 10 years, students attending Greek public schools are expected to have fully acquired spelling strategies and expand on the textual features of various texts and genres (Greek Government Gazette, 2021). Though the expository written discourse development in Greek-speaking children remains underexplored, there is limited evidence showing that it is a dynamic process that develops gradually and is academically attained in adolescence. Specifically, Kantzou’s (2019) study has focused on the syntactic complexity of the expository texts of Greek-speaking children aged 10, 13, and 16 years, and found that children at the age of 10 years are able to produce coherent expository texts, however, they follow a linear organization of information as compared to the older groups that organize their text hierarchically.

Several studies have investigated the spelling error patterns in Greek-speaking children. Across these studies, spelling errors have been classified into three categories, namely, phonological, grammatical and orthographic errors. Phonological errors are defined as those that bring about a change in the phonemic makeup of a word and are mainly derived from grapheme omissions, additions and transpositions (e.g. στ()ατός, instead of στρατός/stratos/‘army’). Grammatical errors are derived from erroneous spelling of inflectional endings that do not, however, distort the phonological identity of the words (e.g. e.g. παίρνι, instead of παίρνει/perni/‘takes’; the spelling error is underlined). Finally, orthographic errors include misspellings of word stems that do not modify the pronunciation of the target word (e.g. ορέος, instead of ωραίος/oreos/‘beautiful’). Protopapas et al. (2013) have examined spelling errors in large samples of 8-, 9-, and 12-year-old children who were tested on word and passage spelling. As expected, 12-year-old children committed considerably fewer spelling errors than the younger groups, indicating that the older group’s system was mature enough to integrate multiple cues to guide spelling processes; however, these mechanisms still undergo development since even 12-year-old children make at least some spelling errors (see also Diamanti et al., 2014 for similar findings). In Protopapas et al.’s (2013) study, there was a significant trend towards fewer phonological errors as compared to grammatical and orthographic errors across all experimental groups, while older children exhibited higher rates of grammatical vs. orthographic errors. Interestingly, phonological and punctuation errors were negligible across all age groups. Diamanti et al.’s (2014) study has also shown significant grammatical category effects on the spelling error patterns of 10–13 year-old Greek-speaking children, since suffixes in nouns were found to be more accurately spelled than stems, yet, spelling errors in the verbs’ suffixes were significantly more than in nouns. This suggests that children’s spelling performance may be explained by processing constraints posited by the syntactic properties of the grammatical categories the words belong to.

Stress assignment constitutes an additional important aspect of writing competence in Greek. Greek is a relatively free-stress language in which the location of stress on a word is restricted to the last three syllables. Researchers (Botinis, 2011; Revithiadou, 1999) have claimed that stress assignment in Greek is planned on the basis of both bottom-up and top-down cues, starting from the segmental phonological properties of the word and spanning the word’s ‘neighborhood’, since stress assignment is also sensitive to the syntactic and prosodic properties of the word’s adjacent items. A characteristic example of the synergy between lexical and syntactic cues in stress assignment is’host-and-clitic’ phrases in Greek, such as [το μάθημά μου]/to_ARTICLE.NEUTER_ máθimá_NOUN.NEUTER_ mu_CLITIC_/‘my lesson’, where the noun appears with two stresses, one on the first syllable and another one on the third syllable of the noun. According to Botinis (2011), the enclitic stress pattern has nothing to do with the enclitic [mu] as such but rather with the phrase boundary. As a core feature of the phonological specification of words, stress assignment is essential in word production. Because of the existence of stress minimal pairs in Greek, such as μόνος/mónos/‘alone’—μονός/monós/‘single’, in which stress alone disambiguates the meaning of the two words in printed text, children can flexibly learn lexical meaning distinctions based on stress assignment, which further contributes to efficient reading performance. In spelling, stress is indicated by a diacritic over the vowel of the stressed syllable, and it is mandatory on every word with two or more syllables. Hence, any omission or misplacement of the stress diacritic is a spelling error.

Despite the importance of stress assignment in both receptive (reading) and expressive written language (spelling), little attention has been paid to the linguistic and cognitive processes involved in lexical stress assignment, the vulnerability of these processes in the case of disorders affecting written language, as well as to the potential usefulness of assessing stress assignment performance for the evaluation of writing competence. In a study of word and pseudoword reading, Protopapas (2006) found that Greek-speaking children made frequent stress assignment errors in pseudowords (i.e. strings of letters that resemble a real word) but not in words, and that such errors were associated with the children’s reading ability, further suggesting that decoding the stress diacritic may be a demanding process and that stress assignment in reading may be largely lexical. Kotoulas and Padeliadu (1999) have examined stress assignment in spelling in Greek and found it to be a highly problematic domain of performance for TD children, while Protopapas et al. (2013) spelling study in 8-to-12 year old Greek-speaking children with and without dyslexia found that the rates of stress assignment errors were comparable to those of orthographic and grammatical spelling errors. Recently, Ralli et al. (2021) found that 8-year-old Greek-speaking children with DLD produced considerably higher rates of errors on the use of the stress diacritic than their TD peers in a short-story writing task. The overall evidence in Greek suggests that stress assignment is a domain of high vulnerability in the writing performance of children with and without disorders. The findings also imply that children’s phonological spelling strategies, which are recruited rather early in spelling development (Porpodas, 2001; Protopapas et al., 2013), may not encompass suprasegmental features, such as stress, at least not beyond the single-syllable level.

## The Current Study

Motivated by previous findings in monolingual individuals with DLD and ASD, we investigated whether written expression may present even more of a challenge to children with DLD and ASD growing up in bilingual settings. In the current study, we examine for the first time the written text production of age-, PIQ-, and SES-matched bilingual Albanian-Greek children with DLD and ASD (along with TD bilingual peers), focusing on both micro-level properties of expository texts that relate to the spelling and stress assignment errors at the word level, and macro-level properties that expand across discourse units of text, such as punctuation errors. We also examine whether the children’s language ability (vocabulary), current language use and home language history measures affect their performance in each writing component.

The children were assessed on two expository essays, which were written by the children in Greek, and were coded for (a) spelling errors (phonological, orthographic, grammatical) on nouns, verbs and adjectives, (b) stress diacritic placement, and (c) punctuation errors. We have focused on 10-to-12 year-old Albanian-Greek children who were exposed to both languages since birth, they were thus simultaneous bilinguals. All bilingual children attended mainstream classes of state schools in central Greece wherein Greek is the only medium of literacy instruction with no heritage language support. This is partially explained by the Albanian parents’ misconception of the children’s home language as an obstacle to the acquisition of the dominant language, i.e. Greek (Gkaintartzi et al., 2015).

The main objective of the current study was to investigate whether spelling errors, stress assignment and punctuation errors differ across DLD, ASD and TD bilingual children. Based on previous research demonstrating several strengths in writing in autism, including the children’s spelling skills (Hilvert, 2018), and robust impairments across all the components of the writing performance in children with DLD (e.g. Graham et al., 2020; Joye et al., 2020), we predicted that the bilingual autistic children’s error rates in spelling would be similar to TD bilingual children and lower than their DLD bilingual peers. Regarding punctuation, based on previous research with individuals with ASD and DLD (Dockrell & Connelly, 2015; Favart et al., 2016; Geelhand et al., 2021), we assumed that both bilingual groups with DLD and ASD would exhibit more punctuation errors than the rest of the groups due to their difficulty in processing mappings between coherent units of meaning and ongoing written discourse.

Furthermore, the study has explored whether the children’s language ability measured through expressive vocabulary, and bilingual experience reflected in the children’s current language use and home language history, would account for the variance in each experimental group’s error rates in spelling, stress placement and punctuation. Deficits on expressive (oral) vocabulary has been proposed to impact a range of writing components, including spelling (e.g. Kim et al., 2013; Santos & Befi-Lopes, 2012) and stress assignment skills (Bellocchi et al., 2016), thus, we expected that the children’s performance in both domains would be affected by their expressive vocabulary performance. Finally, we predicted that the bilingual children’s errors rates in writing would be inversely related to their exposure to Greek, which was the language in which the children were tested.

## Methods

### Participants

The study included eighty-four bilingual children in total, more specifically, 28 TD bilingual children (21 boys; TD, Mean age: 10;5 years, Mean PIQ: 104.5), 28 bilingual children with DLD (23 boys; DLD, Mean age: 10;3 years, Mean PIQ: 107.4), and 28 bilingual children with ASD (24 boys; ASD, Mean age: 10;3 years, Mean PIQ: 110.2). The three groups were matched for chronological age, SES (as indexed by maternal education years) and PIQ using the Wechsler Intelligence Scale-III (Greek version of the Wechsler Intelligence Scale for Children (WISC-III), 3rd edition, Georgas et al., 2003; test–retest reliability of the WISC-III ranged between .94 and .98) (see Table 1). TD bilingual children were recruited from mainstream schools in Greece and they were included in the study if they had no hearing impairments, no speech, emotional or behavior problems, and no neurological or severe articulation/phonological deficits. Their profile was confirmed by information from health screening protocols, which were implemented prior to data collection as part of the Governmental Public Health Policy in Greek public education, and teachers’ and parents’ reports.

**Table 1:** Demographic characteristics of TD bilingual children, and bilingual children with DLD and ASD

	Group means (*SD*) Ranges			*p*^1^	*η*^2^
	TD (n = 28)	DLD (n = 28)	ASD (n = 28)		
Age	10;5 (0.9) 9;10–11;8	10;3 (0.9) 9;10–11;7	10;3 (0.9) 9;1–11;9	.782	.07
PIQ	104.5 (11.6) 89–127	107.4 (16.3) 83–123	110.2 (16.6) 83–132	.373	.15
VIQ	101.7 (10.6) 86–122	67.5 (4.5) 61–69	95.7 (16.9) 80–131	< .001	.78
SES	7.7 (2.1) 6–12	7.9 (2.8) 6–15	8.3 (2.6) 6–13	.655	.10
ADI-R					
Social interaction (cutoff = 10)	–	–	17.8 (1.9) 14–23		
Communication (cutoff = 8)	–	–	11.9 (1.3) 10–15		
Stereotyped patterns (cutoff = 3)	–	–	3.3 (0.7) 3–5		
Current language use (in Greek)	68.9 (8.6) 43–81	66.5 (12.6) 42–86	70.9 (7.1) 54–81	.236	.18
Home language history (in Greek)	64.1 (7.7) 52–78	68.3 (8.1) 54–80	66.4 (8.7) 50–81	.173	.20

*TD* typically-developing bilingual children; *DLD* bilingual children with Developmental Language Disorder; *ASD* bilingual children with Autism Spectrum Disorder; *VIQ* Standard scores from the Verbal IQ scale; *PIQ* Standard scores from the Performance IQ scale; *SES* socio-economic status; *ADI-R* Autism Diagnostic Interview-Revised

^1^Mann–Whitney-U tests

Specifically, bilingual children with DLD were recruited from public diagnostic centers in Greece, which issue a DLD diagnosis based upon the assessment of an inter-disciplinary team of professionals from various disciplines, namely, a speech and language clinician, a clinical psychologist, a teacher specialized in neurodevelopmental disorders, a social worker and a child psychiatrist, child neurologist or developmental pediatrician. According to DSM-5 criteria (American Psychiatric Association, 2013), a diagnosis of DLD was excluded by the presence of any hearing loss, autism, obvious neurological dysfunctions or motor deficits. The diagnosis of DLD was further supported by questionnaires, as well as language and neuropsychological testing. More specifically, parental questionnaires and language unit class teachers’ reports confirmed significant delays in the children’s early language milestones as well as expressive difficulties in both the oral and the written modality (Leonard, 1998). According to the teachers’ academic reports, the majority of the children with DLD faced stronger impairment in the expressive (vs. receptive) modality. Their observations about the children’s language expressive abilities included lexical retrieval delays, reduced speech rate and low frequency of syntactically complex utterances. Moreover, according to the children’s responses to the verbal scales of the WISC-III (Wechsler, 1992; adapted in Greek by Georgas et al., 2003), bilingual DLD children’s verbal abilities were at least two standard deviations (SDs) below the expected normative mean of chronologically age-matched peers, while their non-verbal scores were within the normal limits for their chronological age (i.e., a non-verbal score of 75 or above; Bloom & Lahey, 1978).

Bilingual children with ASD were recruited from schools, public and private diagnostic centers. They had received a diagnosis from a licensed child psychiatrist or developmental pediatrician according to the standard diagnostic criteria, i.e., ICD-10, DSM-IV or DSM-V (American Psychiatric Association, 2013). The severity of the children’s symptoms in the reciprocal social, verbal communication, and stereotyped behavior domains was measured through the Autism Diagnostic Interview-Revised (ADI-R; Rutter et al., 2003; see Table 1). Children with ASD scored considerably above the cut-off on the ADI-R in the social interaction and communication domain, while they marginally exceeded the cut-off score in stereotypies (see Table 1), which implies that the ASD participants faced difficulties with social interaction and communication but exhibited less prevalent stereotyped behavior patterns. Regarding the children’s language profile, the mean verbal IQ score for the ASD group was within the normal range (i.e. VIQ > 80; see Table 1), and it did not differ significantly from their TD peers, but it was significantly higher than DLD children. Similarly, their expressive vocabulary scores were also very close to the TD group (see Table 2) and higher than the DLD group. Moreover, according to the information on the ASD children’s language profiles collected through the teachers’ questionnaires, the children faced pragmatic rather than lexical and morho-syntactic language difficulties, while the parents reported no history of language delay. The reader is reminded that the children with ASD were matched to the other groups on PIQ, which was within the normal range across all the experimental groups (see Table 1).

None of the DLD and ASD bilingual children had received speech and language therapy before inclusion in the study, as data collection took place immediately after receiving the ASD/DLD diagnosis. Though the age of diagnosis for the specific sample of ASD and DLD children is late (Baio et al., 2018), it is not surprising if one considers that a considerable number of children (around 7.100) in Greece in 2019 received a late ASD diagnosis and intervention, at the age of 10 years, which is arguably due to the low primary caregiver’s educational level and high stigma accompanying autism (Thomaidis, 2020). All the children with DLD and ASD attended inclusive classes in schools in which they received literacy skills support in the Language and Mathematics module by a special education teacher. All children with DLD and ASD were included in the study irrespective of whether they presented a writing deficit or not.

The bilingual children across groups came from mixed marriages and they were exposed to both languages from the time of birth, thus they were simultaneous bilinguals. They were Albanian-Greek speakers and they were all dominant in Greek. Children’s bilingual experience was documented through a comprehensive parental questionnaire (see Andreou, 2015 for the questionnaire), which provides information on the quantity of language exposure, operationalized as the relative amount of exposure to each language in various settings and the language(s) used by the child in various daily routines. We should note, however, that exposure and language use were conflated in the questionnaire’s response scales, thus, they were not calculated separately. The bilingual experience for each child was broken up into current and past exposure in each language measured through the current language use and the home language history index, respectively.

The current language use index measures the child’s current language exposure and use of each or both languages (i.e. Albanian and Greek) in oral interactions across different contexts (e.g. daily conversations, helping with the child’s homework, speaking on the phone) with family members (i.e. mother, father, siblings, grandparents and other relatives) and friends. More specifically, a component of the questionnaire included the languages used in interactions with specific interlocutors, who were equally weighted (e.g. “Grandparents/friends talk to the child/the child talks to them mostly in Greek/mostly in Albanian/ in Greek and Albanian equally”). In addition, there was a 3-point scale using quantifying adverbs (i.e. often, sometimes, rarely) that measured language exposure and use in specific activities (e.g. “Mark how often the child watches television programs in Greek/Albanian”). The questionnaire also included time-unit measures asking the parents to report, for instance, on the hours per day that the child spent on the computer hearing or using a language (Greek/Albanian), the duration (in months or years) of the additional lessons the child has taken in Greek or Albanian at home or at school, the frequency of attending these lessons (number of lessons per week and duration), as well as whether these lessons targeted the child’s reading and writing skills. We should note, however, that in the current study, quantity of exposure was based on the parents’ responses in the components of the questionnaire that included the languages used in interactions with specific interlocutors.

The home language history index, on the other hand, measures the bilingual child’s language exposure from birth up to the age of 4 years. Since 2017, compulsory preschool education from 4 years of age has been implemented in all municipalities in Greece. Although recent legislation in Greece provides for the teaching of Greek as a second language to immigrant students, the sole language of formal instruction in state schools is Greek. By obtaining language exposure information up to the age of 4 through the bilingual questionnaire, we aimed to offer a more precise measure of the children’s exposure than just current language use, because home language history quantifies the amount of exposure within a given period of time, up to age 4, i.e. before the bilingual children are immersed in monolingual and monoliterate Greek education. The questions comprising the home language history component asked about the languages that the child heard and used when orally communicating with her/his parents, siblings, grandparents, relatives, and friends before attending kindergarten. The scoring scheme for home language history was similar to that implemented in the current language use component, i.e. an index was calculated on the basis of the languages used in interactions with specific interlocutors. As such, the current language use index provided us with a rather global relative language exposure measure, while home language history yielded a picture of children’s past language experience before entering monolingual/monoliterate preschool education in Greece.

The questions across both the current language use and home language history components of the questionnaire targeted both oral language use and exposure to Greek and Albanian, so quantifying language use and exposure individually was not applicable. Following Kaltsa et al.’s., (2017) scoring of the questionnaire, points were awarded for use of and exposure to each language based on the number of people interacting with the child; for instance, for either current language use or home language history, one or the other language was given 1 point, depending on whether a certain family member (father, mother, siblings, grandparents, etc.) or friend interacted with the child in Greek or Albanian, respectively. If a person interacted with the child in both languages, the point was divided between the two languages (0.5 points each). Current language use/home language history was then quantified via a composite score as a sum of the various language use and exposure experiences that the child had in home and social settings with family members and friends. This score was then normalized (in percentage) for the total number of individuals interacting with the child in one language or the other. For example, when estimating current language use, parents indicated that their child’s language exposure and use with the father, the grandparents, and friends fell within the “Mostly in Greek” range, while language use with the mother and the younger siblings fell within the “Greek and Albanian equally” range. The proportion of current exposure to Greek was calculated as the total number of points awarded for exposure to and use of Greek (in this example, 4 points) divided by the total number of interlocutors (i.e. 5), amounting to 80%. Current exposure to Albanian was calculated in the same way. The sum of current exposure to Greek and Albanian came to 100%. Both current language use and home language history percentages are displayed in Table 1 and correspond to the bilingual child’s language experience in Greek; for example, since the ASD group’s home language history percentage score in Greek was 66.4%, the same group’s home language history score in Albanian would be 33.6%. We should note that the methodology of calculating language exposure in the current study reflects the percentage of interlocutors with whom the children spoke a given language rather than the children’s language experience, i.e. the actual percentage of time during a typical week that the children heard/spoke a given language across numerous interlocutors and settings. Since the current method is not one typically used in bilingualism research (e.g. Thordardottir, 2011, 2015, 2019), its comparability with previous studies with bilingual groups of children may be limited; also, the effect of the children’s bilingual experience on their writing profile might have been different if time-unit measures of bilingual exposure were also taken into account.

Table 1 also presents the chronological ages, verbal and performance IQ as calculated through the WISC-III tool, and the SES of the groups, as well as severity of autism in the reciprocal social, verbal communication, and stereotyped behavior domains as quantified by the ADI-R for the bilingual group with ASD. Continuous data in Table 1 are expressed as means and SDs. Univariate tests were calculated with Mann–Whitney non-parametric tests. There were no significant differences between the groups across the background demographic, the current language use and home language history measures. There was a significant group effect in the VIQ scales of the WISC-III in which the TD and ASD group had significantly better performance as compared to their DLD peers (*p* < 0.001 for both differences); the difference between the TD and ASD children in VIQ was not significant (*p* = 0.138).

Experimental data were collected following all children’s parents’ written consent, children’s assent and obtainment of approval from the Research Ethics Committee of the Greek Ministry of Education and Religious Affairs.

### Measures—General Procedure

All groups of children completed the following tasks in a fixed order: (a) an expressive vocabulary test, and (b) a writing task. As already mentioned, all tasks were administered in Greek. Children were tested individually at school or in a quiet area of their home by the first author. Participants completed the tasks in a single session. Data collection took place over a period of two months (from April 2017 to June 2017).

## Materials and Procedure

### Language Screening—Expressive Vocabulary Task

Children’s expressive vocabulary in Greek was measured through a picture-naming test (Vogindroukas et al., 2009; the Greek version was adapted from Renfrew, 1997). This task is standardized for 3-to-10-year-old Greek-speaking monolingual children, and consists of 50 black-and-white pictures of objects, which are arranged in order of increasing difficulty and which the child was asked to name. Testing stopped when the child either completed all trials or provided wrong naming (or no response) in five consecutive trials. The highest possible score is 50, with each correct naming response earning one point. The test–retest correlation coefficients for the total score ranged between 0.75 and 0.88 across age groups.

### Writing Task

Each child was asked to compose two expository texts on two different topics on a double-sided A4 sheet of paper. The two topics were read aloud to the child by the first author and were also provided on the paper while the child was writing her/his essay: “Describe your neighbourhood” and “Describe your favourite day of the week”. Children were encouraged to write as much as they could, and there was no time or word limit. The choice of the expository writing genre was motivated by the fact that expository texts are introduced from the third grade onwards in the Greek primary educational system, and this genre is well-documented across writing skill workshops that have been recently integrated within the Greek school curriculum. Furthermore, the two topics were among those students frequently engaged with at school or assigned to complete at home.

Coding of text variables. All written texts were coded for spelling, stress and punctuation errors by the first author, and inter-rater reliability checks were conducted by a research assistant blind to the diagnostic groups on 36 (45%) out of the 84 transcripts, which were selected randomly with equal representation of diagnostic criteria (DLD, ASD, TD). Inter-rater reliability in the transcription reached 94.6%, and all discrepancies were resolved through discussion. Furthermore, we calculated the total numbers of each child’s misspellings on content words (i.e. nouns, verbs, and adjectives), as well as counts of content words and verb clauses. The following sections present the way spelling, stress and punctuation errors were coded, along with relevant examples drawn from the children’s expository texts.

Spelling errors. Spelling errors were coded on content words, and they were classified into phonological (see examples 1–3; the spelling errors are underlined), grammatical (see examples 4–6) and orthographic errors (see examples 7–9). The examples below have been derived from the essays of the children that have participated in the current study.

### Phonological Errors

1. δημητ()ιακά, instead of δημητριακά/ðimitriaká/ ‘cereals’
2. δέ()τρα, instead of δέντρα /ðéndra/ ‘trees’
3. πίστες, instead of πίτσες/pìtses/ ‘pizzas’

### Grammatical Errors

(4) λέγετε, instead of λέγεται/léɣete/‘is said’
(5) γονιόν, instead of γονιών/ɣonión/‘parentsGEN’.
(6) τηλεόρασι, instead of τηλεόραση/tileórasi/‘television’

### Orthographic Errors

(7) όμωρφη, instead of όμορφη/ómorfi/‘beatifulFEM/
(8) χοράφια, instead of χωράφια/xoráfjia/‘fields’
(9) γίρισε, instead of γύρισε/ɣìrise/‘returned’

Stress assignment errors. Stress errors were derived from stress diacritic omission and misplacement in words with two or more syllables, as well as from the addition of superfluous stress diacritics besides the target one (see examples 10–12; the stress assignment errors are underlined).

(10) αδελφος, instead of αδελφός/aðelfós/‘brother’
(11) εμείνε, instead of έμεινε/émine/‘stayed’
(12) μέσήμερί, instead of μεσημέρι/mesiméri/‘noun’

Punctuation errors. Based on McCaskill’s (2012) punctuation mark classification system, punctuation errors in the current study consisted of the omission or misplacement of full stops, commas and question marks (see examples 13–15).

(13) Δίπλα στο σπίτι έχει ένα μικρό καφενείο που μερικές φορές μπορείς να

next to-the house has a small café where some times can to

ακούσεις πολλά λαϊκά άσματα έξω από τη γειτονιά μου είναι ένα

listen-to many folk chants out of the neighborhood my is a

ξενοδοχείο

hotel

Next to the house there is a small café where sometimes you may listen to many folk chants. Outside my neighborhood there is a hotel.

(14) το πρωί φεύγω από το σπίτι πάω σχολείο κάνω τα μαθήματα μου παίζω με

the morning leave from the home go school have the lessons my play with

τους φίλους μου και το μεσημέρι γυρίζω σπίτι

the friends my and the noon go-back home

In the morning I leave from home, go to school, have my lessons, play with my friends and at noon I go back home.

(15) τέλος. κάνω τα μαθήματα μου

end. do the homework my

(in the) end. I am doing my homework.

### Analysis Plan

The vocabulary and writing tasks were coded for analysis. All the analyses were performed within the statistical analysis software R (version 3.6.3; R Core Team, 2019). First, we present the results of one-way ANOVA analyses, which were used to compare the groups’ performances in the expressive vocabulary test. Regarding the writing task, the first set of analyses aimed at controlling for potential confounding effects of topic-type (i.e. neighborhood/favorite day) and expository text length, which could have affected the patterns of errors in the children’s written production. We, thus, ran one-way ANOVA analyses to investigate differences in the length of the expository texts across the three experimental groups. As already mentioned, the current design employed two expository topics to prompt writing. To avoid any topic biases, chi-square tests were run comparing raw numbers of content words and verb clauses between the two topics for each of the experimental groups. Furthermore, we ran bivariate correlations between the number of content words and the children’s expressive vocabulary scores in each experimental group to see whether the length of the expository texts correlated with the vocabulary scores.

Considering the heterogeneity in the children’s expository texts and to attenuate the potentially confounding effect of variability stemming from the length of the expository texts and the number of the content words across the experimental groups, percentage scores of each spelling error type were calculated as the total count of a given spelling error divided by the total number of content words. Likewise, percentage total scores of stress errors, as well as scores for each error type, i.e. stress omission, misplacement and superfluous stress addition, were calculated as the counts of stress errors per category divided by the total number of content words (see Protopapas et al., 2013; Ralli et al., 2021). Finally, percentage total scores of punctuation errors, as well as scores for each error type, i.e. punctuation omission and misplacement of punctuation marks, were calculated as the counts of punctuation errors per category divided by the total number of verb clauses (Ferreiro & Pontecorvo, 1999). These percentage scores are presented in the tables of summary statistics alongside the raw counts. Models were created using the percentage scores of the dependent variables.

Separate analyses were conducted for the TD vs DLD, TD vs. ASD, and DLD vs. ASD groups in order to gain full understanding of the way the three writing components, i.e. spelling, stress and punctuation, were affected by each language disorder relative to the TD group, as well as the way in which DLD children differed from their ASD peers in the same writing dimensions. In order to investigate Disorder effects on the error types in the children’s expository texts, logit mixed effects models were performed for each type of error in spelling (i.e. phonological, orthographic, grammatical errors), stress assignment (i.e. stress omission, misplacement and superfluous stress addition errors) and punctuation (i.e. omission and misplacement errors). Disorder (DLD, ASD) was the predictor in each model, while participants were the random slopes. Expressive vocabulary, current language use and home language history were also included as predictors in all models.

## Results

### Language Screening—Expressive Vocabulary

Table 2 below illustrates the groups’ mean scores in the expressive vocabulary task. Group differences in expressive vocabulary were examined through one-way ANOVA analyses. There was a significant Group effect, which stemmed from the fact that the DLD group scored significantly lower relative to both TD and ASD groups (post-hoc Tukey tests; *p* < .001 for both comparisons). There was no significant difference between ASD and TD children (*p* = .998).

**Table 2:** Means and standard deviations (SD) of expressive vocabulary scores across the three experimental groups

	Group means (*SD*)			*p*^1^	*η*^2^
	TD (n = 28)	DLD (n = 28)	ASD (n = 28)		
Expressive vocabulary (max. 50)	36.4 (7.0)	27.9 (9.5)	36.2 (5.6)	< .001	.51

*TD* typically-developing bilingual children; *DLD* bilingual children with Developmental Language Disorder; *ASD* bilingual children with Autism Spectrum Disorder; *n* number; *max* maximum

### Writing Task

To rule out the possibility that there was an effect of topic on the children’s writing performance, we compared the numbers of content words and verb clauses across the two expository texts (*neighbourhood*/*favourite day*) in each experimental group (see Table 3). There was no significant difference in the number of content words between the *Neighborhood* and the *Favorite day* story for either experimental group (χ^2^(1, *N* = 28) = 3.037, *p* = .089 for the TD children; χ^2^(1, *N* = 28) = .244, *p* = .809 for the DLD children; and χ^2^(1, *N* = 36) = 1.134, *p* = .267 for the ASD children). Likewise, there was no significant difference in the number of verb clauses between the two topics for either experimental group (χ^2^(1, *N* = 28) = .460, *p* = .649 for the TD children; χ^2^(1, *N* = 28) = .010, *p* = .989 for the DLD children; and χ^2^(1, *N* = 36) = 1.521, *p* = .218 for the ASD children). Due to the lack of a significant topic effect, the data form the two expository texts were merged before the analyses.

**Table 3:** Means (*SD*) of counts of content words and verb clauses per topic across the three experimental groups

	Group means (*SD*)			*p*^1^	*η*^2^
	TD (n = 28)	DLD (n = 28)	ASD (n = 28)		
Mean N of content words	74.3 (12.8)	57.4 (18.8)	62.1 (26.9)	.008	.34
* ‘Neighborhood’* topic	38.5 (6.7)	28.6 (9.2)	31.5 (13.3)		
* ‘Favorite day’* topic	35.7 (6.6)	28.7 (9.8)	30.5 (13.9)		
Bivariate correlations between mean N of content words and vocabulary (Ν = 28)	.790***	.450*	.419*		
Mean N of verb clauses	32.8 (5.1)	22.7 (11.2)	23.8 (6.8)	< .001	.49
* ‘Neighborhood’* topic	16.5 (3.1)	11.3 (5.5)	12.4 (3.9)		
* ‘Favorite day’* topic	16.3 (3.0)	11.2 (5.8)	11.3 (3.2)		

*TD* typically-developing bilingual children; *DLD* bilingual children with Developmental Language Disorder; *ASD* bilingual children with Autism Spectrum Disorder; *N* number

*p < .05, ***p < .001

Table 3 summarizes the descriptive statistics for the content words and verb clauses in each group’s expository texts. Group differences in content words and verb clauses were examined through one-way ANOVA analyses. There was a significant Group effect in content words, which stemmed from the fact that the DLD group scored significantly lower relative to the TD children (*p* = .007). There was no significant difference either between DLD and ASD children (*p* = .675), or between ASD and TD children (*p* = .069) in content words. Regarding verb clauses, the Group effect was highly significant, and it was due to the fact that both ASD and DLD children used significantly fewer verb clauses than their TD peers (*p* < .001 for both comparisons). There was no significant difference between DLD and ASD children in verb clauses (*p* = .875). Furthermore, Table 3 displays the bivariate correlations between the number of content words and the expressive vocabulary scores in each experimental group. The correlations were significant for all the experimental groups.

Table 4 summarizes the descriptive statistics for the spelling errors per category (i.e. phonological, grammatical and orthographic), stress (i.e. diacritic omission, misplacement, superfluous diacritic addition) and punctuation errors by category (i.e. punctuation marker omission, misplacement). The reader is reminded that spelling and stress error percentages were calculated by dividing the counts of spelling and stress errors by the total number of content words, while punctuation error percentages were calculated by dividing the counts of punctuation errors by the total number of verb clauses.

**Table 4:** Means (SD) of counts and percentage of spelling errors, stress and punctuation errors per error-type across the three experimental groups

	TD (n = 28)		DLD (n = 28)		ASD (n = 28)	
	Counts	Percentage	Counts	Percentage	Counts	Percentage
**Spelling errors**	18.4 (11.2)	26.5 (19.4)	41.3 (20.8)	69.2 (17.7)	26.5 (11.4)	45.7 (18.7)
Phonological	3.0 (2.2)	4.2 (3.0)	3.6 (3.8)	5.8 (5.7)	4.0 (4.1)	7.3 (7.5)
Grammatical	11.7 (8.9)	17.1 (15.6)	22.7 (12.7)	38.4 (14.2)	10.8 (6.7)	19.3 (11.8)
Orthographic	3.7 (3.5)	5.3 (5.2)	15.0 (8.5)	25.1 (9.3)	11.7 (6.9)	19.2 (9.6)
**Stress errors**	6.0 (3.5)	8.5 (5.7)	13.1 (11.0)	24.6 (20.8)	33.6 (21.4)	51.2 (14.9)
Omission	5.3 (3.5)	7.5 (4.3)	11.0 (9.1)	20.7 (16.8)	2.8 (1.7)	4.3 (2.1)
Misplacement	0.5 (0.4)	0.8 (1.2)	1.4 (1.3)	2.7 (3.7)	4.4 (3.9)	6.6 (2.2)
Superfluous addition	0.2 (0.1)	0.2 (0.6)	0.7 (0.5)	1.2 (2.1)	26.4 (17.5)	40.3 (13.7)
**Punctuation errors**	1.2 (1.1)	3.8 (3.4)	5.4 (4.9)	21.3 (15.7)	10.6 (7.8)	43.9 (17.1)
Omission	1.1 (0.9)	3.6 (3.1)	4.9 (3.0)	19.5 (13.6)	2.6 (1.9)	10.7 (6.2)
Misplacement	0.1 (0.1)	0.2 (0.5)	0.5 (0.2)	1.8 (2.3)	8.0 (3.7)	33.2 (14.7)

*TD* typically-developing bilingual children; *DLD* bilingual children with Developmental Language Disorder; *ASD* bilingual children with Autism Spectrum Disorder

### Writing Performance: TD vs DLD

We first investigated the effect of Disorder (DLD, TD) on the bilingual children’s spelling, stress and punctuation errors. Tables 5, 6, 7 display the output of the logit mixed effects models for spelling, stress and punctuation, respectively. The model on spelling showed significant main effects of DLD on overall spelling errors rates (see Table 5). When splitting the spelling errors by type, it was found that the DLD effect mainly stemmed from the fact that the DLD children produced significantly more grammatical and orthographic errors than the TD children. Moreover, the children with low home language history scores, i.e. low exposure to Greek up to the age of 4 years, tended to exhibit more grammatical errors than their peers with high home language history scores. The model on stress did not reveal any DLD effect, however, there were significant effects of vocabulary across all error types, i.e. children with low expressive vocabulary scores tended to omit, misplace or/and add a superfluous stress diacritic on words considerably more often than their peers with high scores in the expressive vocabulary task (see Table 6). Finally, the model on punctuation showed that the children with DLD made significantly more omission and misplacement errors that the TD group, and that the children with low current use of Greek exhibited more misplacement errors than their peers with high current use of Greek (see Table 7).

**Table 5:** Summary of logit mixed effects model: types of spelling errors across TD and DLD children

Predictors	Total spelling errors				Phonological errors				Grammatical				Orthographic			
	Coefficient	SE	z	*p* value	Coefficient	SE	z	*p* value	Coefficient	SE	z	*p* value	Coefficient	SE	z	*p* value
Intercept	30.10	2.93	10.26	.011*	4.62	0.93	4.95	.064	− 27.76	2.00	− 13.87	< .001***	− 15.16	1.01	− 15.01	< .001***
Disorder	− 11.22	3.83	− 2.93	.035*	− 0.43	0.94	− 0.46	.707	− 10.66	2.01	− 5.33	< .001***	− 9.89	1.01	− 9.79	< .001***
Vocabulary	− 0.68	0.57	− 1.19	.335	− 0.14	0.11	− 2.30	.290	− 0.24	0.57	− 0.42	.710	− 0.08	0.14	− 0.57	.569
Current language use	− 0.12	0.27	− 0.47	.672	− 0.07	0.07	− 1.04	.477	− 0.02	0.23	− 0.08	.946	− 0.03	0.13	− 0.20	.863
Home language history	− 0.55	0.31	− 1.78	.333	− 0.09	0.08	− 1.34	.347	− 0.73	0.27	− 2.70	.041*	− 0.10	0.16	− 0.64	.651

*TD* typically-developing bilingual children; *DLD* bilingual children with Developmental Language Disorder; *SE* standard error; Disorder levels: TD vs DLD; Reference level for Disorder: TD

*p < .05, **p < .01, ***p < .001

**Table 6:** Summary of logit mixed effects model: types of stress errors across TD and DLD children

Predictors	Total stress errors				Omission				Misplacement				Superfluous addition			
	Coefficient	SE	z	*p* value	Coefficient	SE	z	*p* value	Coefficient	SE	z	*p* value	Coefficient	SE	z	*p* value
Intercept	− 8.98	2.23	4.03	.072	14.08	3.43	4.09	.004**	2.49	0.84	2.95	.025*	1.09	0.44	2.46	.054
Disorder	− 3.08	1.89	− 1.62	.275	− 6.14	2.95	− 2.08	.08	− 1.70	0.87	− 1.93	.102	− 0.86	0.53	− 1.62	.159
Vocabulary	− 0.08	0.19	− 0.41	.724	− 0.85	0.33	− 7.92	.033*	− 0.21	0.11	− 1.92	.049*	− 0.10	.04	− 2.22	.044*
Current language use	− 0.10	0.11	− 0.96	.464	0.04	0.20	0.19	.850	0.01	0.02	0.06	.959	− 0.02	0.01	− 1.45	.163
Home language history	− 0.07	0.14	− 1.91	.227	0.12	0.24	0.48	.638	0.03	0.04	0.89	.386	0.02	0.03	0.58	.567

*TD* typically-developing bilingual children; *DLD* bilingual children with Developmental Language Disorder; *SE* standard error; Disorder levels: TD vs DLD; Reference level for Disorder: TD

*p < .05, **p < .01, ***p < .001

**Table 7:** Summary of logit mixed effects model: types of punctuation errors across TD and DLD children

Predictors	Total punctuation errors				Omission				Misplacement			
	Coefficient	SE	z	*p* value	Coefficient	SE	z	*p* value	Coefficient	SE	z	*p* value
Intercept	11.18	2.09	5.33	.011*	12.87	2.04	6.29	< .001***	1.21	0.34	3.52	.003**
Disorder	− 7.66	1.88	− 4.07	.018*	− 9.20	1.99	− 4.60	< .001***	− 1.01	0.34	− 2.91	.011*
Vocabulary	− 0.09	0.21	− 0.45	.658	− 0.46	0.23	− 1.97	.114	0.01	0.06	0.03	.976
Current language use	− 0.26	0.15	− 1.74	.048*	− 0.31	0.22	− 1.45	.166	− 0.06	0.02	− 2.70	.019*
Home language history	− 0.34	0.21	− 1.64	.264	− 0.16	0.21	− 0.81	.427	− 0.03	0.02	− 1.05	.301

*TD* typically-developing bilingual children; *DLD* Developmental language disorder; *SE* standard error

Disorder levels: TD vs DLD; Reference level for Disorder: TD

*p < .05, **p < .01, ***p < .001

### Writing Performance: TD vs ASD

We next investigated the effect of Disorder (ASD, TD) on the bilingual children’s spelling, stress and punctuation errors. Tables 8, 9, 10 display the output of the logit mixed effects models for spelling, stress and punctuation, respectively. The model on spelling showed a significant main effect of ASD on orthographic errors only (see Table 8). Furthermore, vocabulary was found to be significantly inversely correlated with the children’s orthographic errors, meaning that that the children with low expressive vocabulary scores tended to make more orthographic errors in their writing as compared to their peers with high scores in the expressive vocabulary test. Regarding stress, there was a significant ASD effect which stemmed from the fact that the group with ASD made more misplacement and superfluous addition errors than their TD peers (see Table 9). Finally, in punctuation, there was a significant ASD effect which stemmed from the fact that the children with ASD made more punctuation misplacement errors than the TD group (see Table 10).

**Table 8:** Summary of logit mixed effects model: types of spelling errors across TD and ASD children

Predictors	Total spelling errors				Phonological errors				Grammatical				Orthographic			
	Coefficient	SE	z	*p* value	Coefficient	SE	z	*p* value	Coefficient	SE	z	*p* value	Coefficient	SE	z	*p* value
Intercept	23.26	2.25	10.33	.009**	5.70	0.76	7.48	< .001***	− 18.17	1.86	− 9.77	< .001	− 13.19	1.79	− 7.36	.012*
Disorder	− 4.55	1.91	− 2.37	.161	− 1.57	0.93	− 1.68	.222	− 1.08	1.86	− 0.58	.564	− 8.43	2.21	− 3.81	.032*
Vocabulary	− 1.04	0.38	− 2.79	.190	− 0.22	0.16	− 1.44	.260	− 0.76	0.35	− 2.19	.043*	− 0.25	0.16	− 1.61	.031*
Current language use	− 0.29	0.21	− 1.41	.239	− 0.01	0.11	− 0.05	.066	− 0.35	0.25	− 1.41	.206	− 0.18	0.14	− 1.25	.285
Home language history	− 0.04	2.12	− 0.19	.880	− 0.09	0.10	− 0.90	.420	− 0.60	0.25	− 2.44	.046*	− 0.09	0.12	− 0.72	.488

*TD* typically-developing bilingual children; *DLD* bilingual children with Developmental Language Disorder; *SE* standard error; Disorder levels: TD vs DLD; Reference level for Disorder: TD

*p < .05, **p < .01, ***p < .001

**Table 9:** Summary of logit mixed effects model: types of stress errors across TD and ASD children

Predictors	Total stress errors				Omission				Misplacement				Superfluous addition			
	Coefficient	SE	z	*p* value	Coefficient	SE	z	*p* value	Coefficient	SE	z	*p* value	Coefficient	SE	z	*p* value
Intercept	− 19.80	2.05	9.65	< .001***	5.89	0.45	12.99	< .001***	3.86	0.30	12.88	< .001***	20.53	1.68	12.18	< .001***
Disorder	− 13.80	2.05	− 6.72	< .001***	− 0.59	0.35	− 1.52	.111	− 3.05	0.31	− 10.11	< .001***	− 20.31	1.69	− 12.04	< .001***
Vocabulary	− 0.70	0.34	− 2.08	.023*	− 0.12	0.09	− 1.40	.023	− 0.01	0.09	− 0.10	.927	− 0.02	0.56	− 0.04	.972
Current language use	− 0.04	0.31	− 0.14	.901	0.03	0.07	0.39	.747	0.06	0.05	1.13	.417	0.09	0.38	0.24	.888
Home language history	− 0.46	0.27	− 1.74	.229	− 0.15	0.06	− 2.51	.042*	− 0.02	0.05	− 0.39	.706	− 0.56	0.33	− 1.68	.104

*TD* typically-developing bilingual children; *ASD* bilingual children with Autism Spectrum Disorder; *SE* standard error

Disorder levels: TD vs ASD; Reference level for Disorder: TD

*p < .05, **p < .01, ***p < .001

**Table 10:** Summary of logit mixed effects model: types of punctuation errors across TD and ASD children

Predictors	Total punctuation errors				Omission				Misplacement			
	Coefficient	SE	z	*p* value	Coefficient	SE	z	*p* value	Coefficient	SE	z	*p* value
Intercept	5.89	0.75	7.83	< .001***	5.61	1.46	3.82	.015*	16.56	1.84	8.98	< .001***
Disorder	− 4.68	0.75	− 6.22	< .001***	− 2.56	− 1.10	2.32	.102	− 16.39	1.83	− 8.91	< .001***
Vocabulary	− 0.24	0.14	− 1.68	.260	− 0.01	0.13	− 0.02	.990	− 0.23	0.48	− 0.49	.653
Current language use	− 0.13	0.11	− 1.21	.368	− 0.11	0.09	− 1.33	.219	− 0.13	0.33	− 0.38	.758
Home language history	− 0.21	0.11	− 2.05	.036*	− 0.18	0.11	− 1.65	.121	− 0.43	0.30	− 1.44	.165

Disorder levels: TD vs ASD; Reference level for Disorder: TD

*TD* typically-developing bilingual children; *ASD* bilingual children with Autism Spectrum Disorder; *SE* standard error

*p < .05, **p < .01, ***p < .001

### Writing Performance: DLD vs ASD

We finally investigated Disorder-type (DLD vs ASD) effects on the bilingual children’s errors. Tables 11, 12, 13 display the output of logit mixed effects models for spelling, stress and punctuation, respectively.The mixed effects model on spelling showed that the children with DLD produced significantly more grammatical errors than their ASD peers (see Table 11). Regarding stress, there was a main effect of Disorder which stemmed from the fact that the children with DLD tended to omit the stress diacritic more frequently than the group with ASD, while the group with ASD tended to make stress misplacement and superfluous addition errors significantly more frequently than their peers with DLD (see Table 12). Finally, in punctuation, there was a significant main effect of Disorder which stemmed from the fact that the group with DLD made more omissions than their ASD peers, while the group with ASD made more misplacement errors than the group with DLD (see Table 13). Also, punctuation omissions were found to be inversely correlated with the participants’ current language use scores; thus, the children with high current use in Greek tended to omit punctuation markers less frequently than their peers with low scores.

**Table 11:** Summary of logit mixed effects model: types of spelling errors across DLD and ASD children

Predictors	Total spelling errors				Phonological errors				Grammatical				Orthographic			
	Coefficient	SE	z	*p* value	Coefficient	SE	z	*p* value	Coefficient	SE	z	*p* value	Coefficient	SE	z	*p* value
Intercept	33.46	2.55	13.08	< .001***	6.59	1.09	6.01	.014*	− 16.75	1.35	− 12.33	< .001***	− 13.37	1.03	− 12.96	< .001***
Disorder	− 6.54	4.27	− 1.53	.242	− 0.80	1.11	− 0.73	.537	− 5.96	1.35	− 4.39	< .001***	− 1.36	1.47	− 0.92	.460
Vocabulary	− 0.36	0.41	− 0.86	.467	− 0.11	0.17	− 0.64	.557	− 0.23	0.29	− 0.79	.523	− 0.18	0.18	− 1.00	.406
Current language use	− 0.02	0.27	− 0.07	.948	− 0.05	0.11	− .44	.670	− 0.02	0.17	− 0.11	.941	− 0.06	0.14	− 0.40	.708
Home language history	− 0.22	0.47	− 0.47	.672	− 0.03	0.11	− 0.23	.848	− 0.26	0.19	− 1.38	.352	0.03	.14	− .223	.834

Disorder levels: TD vs ASD; Reference level for Disorder: TD

*TD* typically-developing bilingual children; *ASD* bilingual children with Autism Spectrum Disorder; *SE* standard error

*p < .05, **p < .01, ***p < .001

**Table 12:** Summary of logit mixed effects model: types of stress errors across DLD and ASD children

Predictors	Total stress errors				Omission				Misplacement				Superfluous addition			
	Coefficient	SE	z	*p* value	Coefficient	SE	z	*p* value	Coefficient	SE	z	*p* value	Coefficient	SE	z	*p* value
Intercept	− 36.14	4.37	8.27	.009**	12.03	4.86	2.47	.062	4.56	0.56	8.12	< .001***	21.00	1.73	12.13	< .001***
Disorder	14.21	2.43	5.83	< .001***	− 7.74	4.36	− 1.77	.044*	1.89	0.46	4.11	< .001***	19.81	1.65	12.01	< .001***
Vocabulary	− 0.06	0.45	− 1.38	.417	− 0.52	0.33	− 1.58	.266	− 0.12	0.06	− 1.82	.083	− 1.18	0.39	− 3.02	.074
Current language use	− 0.08	0.31	− 0.26	.814	− 0.01	0.21	− 0.04	.971	0.08	0.05	1.54	.316	− 0.42	0.29	− 1.40	.181
Home language history	− 0.47	0.29	− 1.61	.045*	− 0.26	0.25	− 1.03	.370	− 0.04	0.06	− 0.68	.532	− 0.09	0.40	− 0.22	.854

Disorder-type levels: DLD vs ASD; Reference level for Disorder: DLD

*DLD* bilingual children with Developmental Language Disorder; *ASD* bilingual children with Autism Spectrum Disorder; *SE* standard error

*p < .05, **p < .01, ***p < .001

**Table 13:** Summary of logit mixed effects model: types of punctuation errors across DLD and ASD children

Predictors	Total punctuation errors				Omission				Misplacement			
	Coefficient	SE	z	*p* value	Coefficient	SE	z	*p* value	Coefficient	SE	z	*p* value
Intercept	29.51	3.84	7.67	.001**	15.80	2.05	7.68	< .001***	17.30	1.83	9.42	< .001***
Disorder	11.27	10.93	5.09	< .001***	− 6.80	2.67	− 2.54	.036*	15.60	1.86	8.36	< .001***
Vocabulary	− 0.54	0.37	− 2.44	.036*	− 0.92	0.30	− 2.71	.085	− 0.96	0.33	− 2.84	.082
Current language use	− 0.55	0.27	− 2.07	.049*	− 0.35	14.87	− 2.29	.037*	0.19	0.27	0.69	.492
Home language history	− 0.67	0.27	− 3.57	.020*	0.05	17.24	0.28	.784	− 0.15	0.33	− 0.46	.666

Disorder-type levels: DLD vs ASD; Reference level for Disorder: DLD

*DLD* bilingual children with Developmental Language Disorder; *ASD* bilingual children with Autism Spectrum Disorder; *SE* standard error

*p < .05, **p < .01, ***p < .001

## Discussion

The current study investigated the writing abilities of bilingual children with DLD and ASD, along with their TD bilingual peers, with the aim of probing specific dimensions that would differentiate the DLD and ASD groups from TD children, as well as the two groups with DLD and ASD themselves. The study also set out to examine the effect of the current language use and home language history measures, as well as the role of language ability in the writing performance of the three groups and, thus, shed light on the mechanisms that could serve as potential factors regulating the children’s text production. The results of the study revealed discrepancies between the experimental groups across the various writing components that have been tested. More specifically, both bilingual groups with DLD and ASD exhibited more spelling and punctuation errors relative to their TD peers, yet, the writing profile of ASD children was found to be less severely affected than DLD children at least at the level of spelling content words. On the other hand, stress diacritic assignment and punctuation errors appeared to be the hallmark characteristic of ASD children’s textual output, since the latter group tended to misplace stress diacritics and punctuation markers, as well as inappropriately use superfluous stress diacritics on lexical items to a significantly greater extent as compared to both their TD and DLD peers. The results also suggest associations between the DLD and ASD groups’ punctuation errors and language exposure measures, while spelling and stress errors were rather associated with vocabulary knowledge for both groups with DLD and ASD. The overall evidence suggests the promising role of writing in the study of the cognitive profile of bilingual children with neurodevelopmental disorders. At the same time, the results highlight writing components that may serve as markers of ASD and DLD in school-aged bilingual children.

The objective of the study was to identify patterns of spelling, stress assignment and punctuation errors that would uniquely characterize each experimental group. Regarding overall spelling errors, the DLD group exhibited the most erroneous performance across the experimental groups, which aligns with developmental literature showing that language is the locus of predominant impairment in DLD (e.g. Leonard, 2014; Van der Lely, 1997). This finding seems to also agree with Ralli et al.’s (2021) study that found the spelling errors rates of Greek-speaking monolingual children with DLD to be considerably higher relative to their TD peers; however, information on the type(s) of spelling errors that drove the group effect is lacking in Ralli et al.’s (2021) study. In the current research, there was a predominance of grammatical and orthographic errors across the three experimental groups, and a very small proportion of phonologically implausible errors types. This suggests that all three groups tended to employ the developmentally sophisticated strategy of using phoneme-to-grapheme correspondences when attempting to spell words in their expository essays. Protopapas et al. (2013) have also reported a negligible proportion of phonological errors in their study of the spelling performance of 8-, 9-, and 12-year-old Greek-speaking monolingual children with and without dyslexia. The overall evidence indicates phonographemic mapping as a systematic and robust spelling strategy across the three groups of children that spoke languages with relatively transparent orthographies.

The experimental groups’ error rates in spelling seemed to diverge with grammatical and orthographic errors. More specifically, the DLD group exhibited higher rates of grammatical errors, i.e. erroneous spellings of the words’ inflectional endings without concurrent distortion of the phonological identity of the word, than their ASD and TD peers. Previous research (e.g. Goodwin et al., 2013; Larkin et al., 2013; Mackie & Dockrell, 2004) has shown that encoding inflectional endings in the written modality constitutes a considerably vulnerable domain in DLD, which could be attributed to a deficit in the children’s rule-based processing for inflectional markers. We also found that the DLD group’s grammatical error rates were modulated by the children’s home language history scores, meaning that the bilingual children that had more exposure in Greek at home up to the age of 4 years tended to make fewer errors in inflectional suffixes in writing as compared to the children with less exposure in Greek. This finding suggests that the amount of exposure in Greek in the early years affected bilingual DLD children’s grammatical awareness and morphological processing, as correct spelling of suffixes in Greek requires knowledge of inflectional morphology. This effect demonstrates that early oral exposure and use of Greek can add to the spelling skills of bilingual children with grammatical deficits.

Interestingly, the children with ASD did not differ from their TD peers in grammatical errors. This finding disagrees with Hilvert’s (2018) study, in which monolingual English-speaking children with ASD made significantly more grammatical errors in their expository essays than their TD peers. Besides testing monolingual autistic children, Hilvert’s (2018) coding design differs from the current study, since it targeted a considerably wider range of grammatical errors, including omitted obligatory tense markers, missing grammatical morphemes on nouns, wrong forms of verbs, and pronoun number or case errors, among others. Furthermore, children’s grammatical errors in Hilvert’s (2018) study mainly consisted of inflectional morpheme omissions which cannot be observed in the data of the current study, since, in a language like Greek, it is only after the affixation of the inflectional suffix that the root can be realized as a word. The finding that the ASD children in the current study did not diverge from their TD peers in grammatical spelling implies that rule-based processing for inflectional endings in writing was a relatively cost-free process for them. The fact that spelling inflectional suffixes was not challenging for the ASD group may be attributed to autistic traits, and more specifically, to autistic individuals’ high ability to engage in systemizing thinking (Baron-Cohen et al., 2009; Smith et al., 2010), which is characterized by a drive towards understanding and predicting rule-based systems that could potentially have boosted the spelling of inflectional suffixes. An alternative explanation for the ASD group’s error-free performance in suffix spelling is that these children had intact language skills which allowed them to compute the words’ grammatical endings correctly. Evidence in favor of the fact that the children with ASD had preserved language competence is that both their verbal intelligence and vocabulary scores were within the normal range and did not differ significantly from their TD peers. The fact that correct spelling of word stems cannot be predicted through rule inferencing but requires root-specific knowledge or/and skilled memory retrieval of the stems’ orthographic representation may be one of the reasons why the ASD group made significantly more orthographic errors than their TD peers. Importantly, orthographic error rates in the ASD and TD groups were significantly associated with the children’s expressive vocabulary skills, which further corroborates the relation between oral language skills and the maturity of the developing orthographic lexicon. This hypothesis proposes that the acquisition of a larger vocabulary boosts the development of phonological awareness by increasing children’s sensitivity to small changes in the words’ sound segments; enhanced phonological awareness in turn facilitates spelling (Metsala, 1999; Wang et al., 2013). The relation between oral language and written production also receives support from the fact that the children’s expressive vocabulary scores were found to be positively correlated with the number of content words in the expository texts of each experimental group. Overall, the significant correlations between spelling, vocabulary and exposure to the dominant language (i.e. Greek) across groups suggest that the differences between TD and ASD, as well as DLD children in the current study are unlikely to be explained by a possible negative influence of bilingualism in children, but rather by language exposure and vocabulary factors.

Stress diacritic placement, along with punctuation errors, appeared to be the hallmark features in the ASD group’s writing profile. Specifically, diacritic placement in roughly half of the content words in ASD children’s expository essays was erroneous (see Table 4). According to the error type analysis, the majority of the errors for the ASD group involved stress misplacement or/and inappropriate placement of more than one diacritic on more than one vowel of two-, three-, four- and five-syllable words. As such—and in spite of the fact that stress on a phonological word in Greek is restricted to the last three syllables—there were instances of written words with antepenultimate and anteantepenultimate stress in ASD children’s writing. According to the results, stress misplacement and superfluous diacritic addition errors for the group with ASD were significantly more than their TD and DLD peers. The DLD children, on the other hand, have made more stress omissions than TD and ASD children, and thus, DLD children’s divergence from the TD group was less striking as compared to the ASD group. DLD children’s use of stress in writing was significantly related to their vocabulary scores, which means that stress errors were mainly committed by children with low oral lexical skills. This seems to align with previous research claiming that the most important source of stress assignment information for Greek is lexical, and that lexical cues have a major contribution to children’s ability to detect the location of stress in a word (Protopapas, 2006; Grimani & Protopapas, 2017).

The evidence on stress errors suggests that stress diacritic placement was a persistent problem for DLD, and especially for ASD children, which is surprising given that the stress diacritic and its obligatoriness are taught at Greek schools as part of regular reading instruction from the beginning of first grade. The difference in the stress error pattern between the group with DLD and the group with ASD, i.e. prominent stress diacritic omission for the DLD children, and misplacement or/and superfluous diacritic misuse for the ASD children, speaks in favor of the view that stress assignment may be derived from information beyond the boundaries of the individual word. As already mentioned, stress assignment in Greek can be influenced by both the words’ segmental phonological properties and the phonological properties within the scope of the word’s adjacent items (Botinis, 2011; Revithiadou, 1999). If stress assignment operates at both the word and phrase level, one may assume that learning how to correctly use stress in writing is adaptive to lexical, syntactic and prosodic constraints which would increase demands for the ASD children whose language deficits go beyond the lexical domain. Future work should further investigate the manipulation of stress in monolingual and bilingual child populations with DLD and ASD to shed more light on the locus of impairment that underlies their erroneous performance.

Finally, both DLD and ASD groups exhibited significantly higher error rates in punctuation than their TD peers, yet, the two clinical groups selectively struggled with distinct aspects of punctuation use. More specifically, the children with DLD tended to omit punctuation markers considerably more than TD and ASD children, while the group with ASD exhibited significantly more extensive misuse of full stops, question marks and commas in their expository essays than the rest of the experimental groups. As already mentioned, punctuation in writing serves a pragmatic function since it contributes to the chunking of text into coherent units on the basis of syntactic and discourse knowledge, and thus aids the extraction of pragmatically felicitous meaning. The appropriate use of punctuation shows that an individual has good syntactic parsing and pragmatic abilities, and it helps to bring thought into writing for which voice, intonation, volume, tone, pauses are used while speaking (Ferreiro & Pontecorvo, 1999). We assume that the inappropriate use of punctuation marking, which was a hallmark feature of ASD children’s performance in the current study, reflects a more deviant or improper implicit prosodic marking in written production as compared to punctuation omission, and that this pattern may be attributed to pragmatic deficits, which have been widely acknowledged to characterize autistic children’s language skills, or/and to ASD children’s putting less weight on implicit prosodic cues to convey meaning in the essay (Durrleman & Delage, 2016; Eigsti et al., 2007; Walenski et al., 2006). Interestingly, both DLD and ASD children’s punctuation performance correlated with their current language use scores, further suggesting that language exposure and use of Greek over time has significantly modulated the children’s manipulation of implicit prosodic cues in their expository essays.

The overall evidence of the study confirms that writing is more challenging for DLD and ASD children relative to their TD peers at several levels of the writing process, including spelling, stress and punctuation. Crucially, the children’s error rates and patterns across the various writing constructs were able to distinguish between the DLD and the ASD group. Specifically, DLD children exhibited greater vulnerability in spelling as opposed to ASD children who were more erroneous in stress and punctuation. Crucially, omission of stress and punctuation was the dominant error type for DLD children, while the children with ASD tended to use stress diacritics and punctuation markers in their writing, but were prone to either misplacing or/and overusing them, which further implies that the children with ASD possessed basic knowledge of suprasegmental features but could not integrate them in writing. The findings suggest that the deficits underlying the writing skills of the DLD and ASD groups may be distinct—for example, that the writing performance of the ASD group is rather affected by deficits that span discourse units rather than single words, while DLD children’s writing deficits are rather reflected in low-level properties of the text, such as spelling.

Turning to the implications of this research, these can be considered at two levels. From a theoretical perspective, the findings may inform hypotheses about the mechanisms underlying writing performance in bilingual children diagnosed with DLD and ASD, and allow us to explain the specificities of spelling, stress and punctuation errors across the two disorders. More specifically, the present study provides evidence that error types in writing can distinguish the deficits of autistic bilingual children from those of children with DLD or/and their TD peers, mainly by being based on stress and punctuation use. Crucially, the group with ASD tended to use superfluous stress diacritics and punctuation in words and discourse units, respectively, considerably more than the rest of the experimental groups, while the bilingual group with DLD faced difficulty mainly with spelling. The specific patterns suggest that the deficits in ASD and DLD surface at distinct levels of writing, namely, at the word and the discourse organization level, respectively. At a more practical level, examining writing skills is important to develop appropriate, targeted writing interventions across the school-age years that meet the literacy needs of bilingual children with DLD and ASD. As such, the findings of the current study may be beneficial to educators developing and implementing instructional practices to support writing and broader literacy development in the specific populations.

The current study has four limitations. First, because of the limited number of standardized language ability tests in Greek, it is unknown whether a more fine-grained approach to the children’s language profile would reveal more associations between language skills and the children’s writing performance. Second, the small sample size of the children that have participated in the current study may have limited the statistical power of the results. Third, the children’s bilingual experience could have been more reliably reflected through calculating the actual percentage of time during a typical week that the children heard/spoke a given language across numerous interlocutors and settings, rather than through computing the percentage of interlocutors with whom the children spoke the two languages. The fact that language exposure and use were conflated in the bilingual questionnaire, and that time-based measures of language exposure at home and at school were not calculated, did not allow us to scrutinize the effect of each factor on the bilingual children’s writing performance; also, the lack of weighting across the various interlocutors the bilingual children interacted with, prevented us from assessing their loading on the participants’ bilingual experience. Finally, more measures which include theory-of-mind and executive functions could have shed more light on the evaluation of the bilingual ASD and DLD children’s writing performance. Therefore, further studies are warranted to investigate the extent to which the children’s writing performance is affected by their executive functions and theory-of-mind skills, as well as how their writing skills might differ across writing genres. In fact, the current research is followed-up by a study of the same children’s writing skills in personal, autobiographical topics, and the preliminary evidence is promising in highlighting significant effects of writing genres on DLD and ASD children’s spelling, stress and punctuation errors.
